# Design and Fabrication by Thermal Imprint Lithography and Mechanical Characterization of a Ring-Based PDMS Soft Probe for Sensing and Actuating Forces in Biological Systems

**DOI:** 10.3390/polym11030424

**Published:** 2019-03-05

**Authors:** Tommaso Dattoma, Antonio Qualtieri, Gianmichele Epifani, Massimo De Vittorio, Francesco Rizzi

**Affiliations:** 1 Center for Bio-Molecular Nanotechnologies@Unile, Istituto Italiano di Tecnologia, Via Eugenio Barsanti 14, 73010 Arnesano (LE), Italy; tommaso.dattoma.1984@gmail.com (T.D.); antonio.qualtieri@iit.it (A.Q.); massimo.devittorio@iit.it (M.D.V.); 2 Istituto di Nanotecnologia, Consiglio Nazionale delle Ricerche, 73100 Lecce, Italy; gianmichele.epifani@nanotec.cnr.it; 3 Dipartimento di Ingegneria dell’Innovazione, Università del Salento—Complesso Ecotekne, edificio “Corpo O”—Via per Monteroni, 73100 Lecce, Italy

**Keywords:** PDMS, thermal imprint lithography, soft probe, force sensor, hair cell, mechanosensing

## Abstract

In this paper, the design, fabrication and mechanical characterization of a novel polydimethylsiloxane (PDMS) soft probe for delivering and sensing forces in biological systems is proposed. On the basis of preliminary finite element (FEM) analysis, the design takes advantage of a suitable core geometry, characterized by a variable spring-like ring. The compliance of probes can be finely set in a wide range to measure forces in the micronewton to nanonewton range. In particular, this is accomplished by properly resizing the ring geometry and/or exploiting the mixing ratio-based elastic properties of PDMS. Fabrication by the thermal imprint lithography method allows fast and accurate tuning of ring sizes and tailoring of the contact section to their targets. By only varying geometrical parameters, the stiffness ranges from 1080 mNm^−1^ to 50 mNm^−1^, but by changing the base-curing agent proportion of the elastomer from 10:1 to 30:1, the stiffness drops to 37 mNm^−1^. With these compliances, the proposed device will provide a new experimental tool for investigating force-dependent biological functions in sensory systems.

## 1. Introduction

In biology, many processes at the molecular, cellular or tissue level are regulated by complex force-dependent mechanisms [1]. For example, many biological processes, such as cellular adhesion [2], motility [3] and transport [4], are promoted by mechanotransduction, which converts external stimulations into biochemical and biological responses [5]. Essential physiological functions, including the sense of touch and hearing, are elicited by this mechanism [6] through highly specialized mechanoreceptors, such as hair cells in the inner ear cochlea, which are responsible for the sensitivity and selectivity of hearing [7,8,9]. Understanding how biological functions are related to cellular mechanics closely follows technological progresses, which provide experimental tools for manipulation and monitoring [10], including sensory systems. Biosensors of different technology convert a biological response from one form to another, specifically optical [11], electrical [12,13], thermal [14] and mechanical [15,16], in dependence with the measurable output. In particular, a mechanical biosensor provides a force readout on the basis of the compressive (tensile) [10] or bending [17] deformation of its most compliant component. As a conversion gain, the stiffness of the sensor, which is comparable to the sensing element for a proper behavior [18], transduces the force to be detected into a quantifiable displacement [19], in accordance with Hooke’s law. In typical applications, mechanical biosensors are requested to transduce forces down to the femtonewton (10^−15^ N) scale and displacements of few angstroms (10^−10^ m) [20] for studying molecular interactions, and even up to micronewtons (10^−6^ N) [21,22] and micrometers (10^−6^ m) [23] for cell and tissue investigations. Additionally, for reliable sensing, the transducer is requested to mimic the in vivo mechanism of stimulation by perceiving and delivering localized stimuli in a controlled way while securely contacting the specific geometry of the sensing element [24]. Finally, delivery or sensing methods should allow the visual accessibility of the cell or tissue for the observation of response. For instance, in the hearing organ, biosensors are used for the investigation of some critical aspects of mechanotransduction, such as the hair cells contribution to amplification and frequency selectivity [25]. For this purpose, sensory devices are needed to convert forces in the piconewton (10^−12^ N) range [26] (stiffness of few mNm^−1^), at frequencies up to some tens of kilohertz [27], at a microsecond time resolution [28], while uniformly matching the unique bundle-based arrangement of cilia in the cochlea.

Methods for inspecting cells and tissues include optical [29] and magnetic [30] tweezers, atomic force microscopy (AFM) probes [31], flexible glass fibers [32,33] and micro-cantilevers [23]. However, the requisites of high compliance and contact control are not simultaneously achieved in any of these instruments. Optical and magnetic tweezers are capable of measuring minuscule forces, down to ~10^−13^ N [34], with a stiffnesses of ~10^−2^ mNm^−1^ [35] and ~10^−6^ mNm^−1^ [34], respectively, but the contact geometry is spherical, and the cellular loading is non-uniform throughout the measurement. For larger forces, up to some nanonewtons (10^−9^ N) [35], AFM probes are used, by taking advantage of a stiffness in the range 10–10^5^ mNm^−1^ [34]. However, measurements are only taken vertically through a random and inhomogeneous contact. In addition, custom-designed fibers in glass or silicon, which are very stiff materials, can have compliances <10 mNm^−1^ [23,24,25,26,27,28,29,30,31,32,33,34,35,36] for mm-long structures, but their circular or pointed contact area [23] is not ideal for all applications. The contact geometry is not improved and is also still random in smaller piezoresistive cantilevers, as reported in [18,19,20,21,22,23,24,25,26,27,28,29,30,31,32,33,34,35,36,37].

Soft materials with high compliance and ease of shape design, for improving the contact geometry with the cell, are needed in order to design a probe with suitable properties to engage cells with a comparable stiffnesses and forces in the pN to μN range. The mechanical properties of the exploited material for probes is critical: A probe’s compliance has to be equal to or less than the cells (10 mNm^−1^). A low Young modulus (~1 MPa), typical of elastomers, is needed. Polydimethylsiloxane (PDMS), by virtue of its excellent physical properties, is a good candidate. Moreover, it has a good thermal stability, low surface tension and good transparency, properties needed to exploit PDMS with thermal imprint lithography (TIL) [38], a microlithography technique with a high throughput, an ultra-high resolution and low cost. This technique allows the fabrication of miniaturized polymeric-based devices. The basic process consists of imprinting a pattern through a master stamp in a liquid polymer thin layer by applying a certain pressure, followed by the polymeric curing process (by heating or UV curing), obtaining an impression of the stamp inside the polymer. This technique allows the technological limits of conventional probes to be overcome by fabricating probes in a polydimethylsiloxane (PDMS) soft polymer, which are characterized by good elasticity, because PDMS exists in a highly coiled conformers, serving well in device applications where the structural material should be bended or twisted. Moreover, PDMS’s inertness, stability, flexibility and non-fluorescent properties are crucial from a biomedical application point-of-view [39].

Herein, the fabrication, processing and characterization of a new spring-like polydimethylsiloxane (PDMS) force probe for investigating forces and mechanical properties of cellular systems is proposed. In particular, the compliance of the probe, working both as a force sensor and force actuator, is finely tuned in a wide range by exploiting the elastic properties of PDMS, which are tuned by differentially dosing the pre-polymer and curing agent ratio, and a proper design of the core structure. Moreover, the customized tip geometry allows for the best fitting and adhesion with a variety of biological sensory receptors to experimentally replicate in vivo stimulation and improve the reliability of measurements. Without a loss of generality, in this paper, the contact geometry suits the inner ear cellular receptor, however, it can be modified depending on the application.

## 2. Materials and Methods

**Figure 1:**
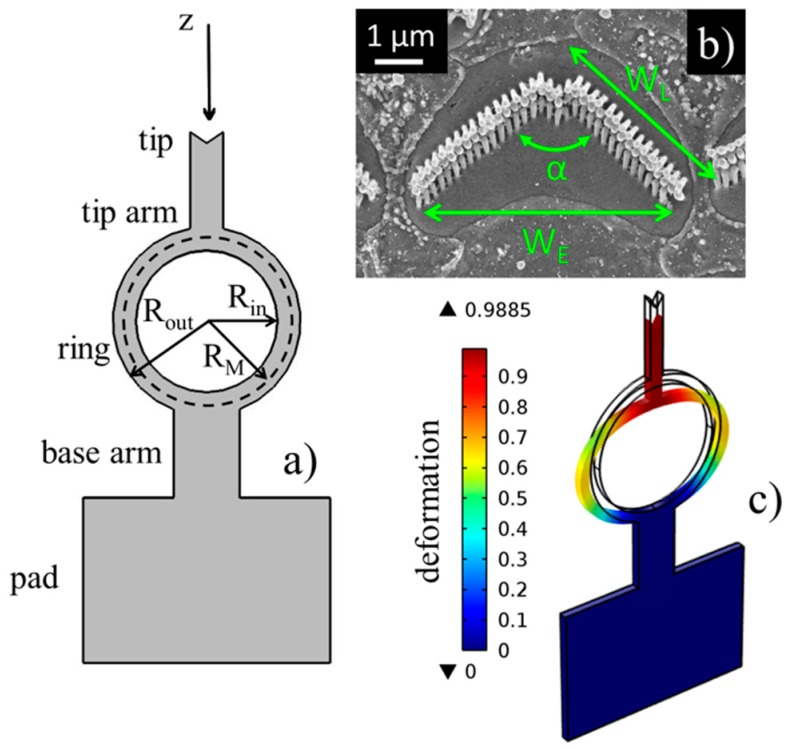
(**a**) Schematic view of the polydimethylsiloxane (PDMS) soft probe. The ring is the most compliant component of the probe, which is properly tuned to provide the final stiffness of the device. The V-shaped tip of the probe is tailored to fit a cochlear bundle of cilia in a hear cell. (**b**) Hair cell bundle in the cochlea (scale bar: 1 µm). Reproduced from Dattoma et al. [40] with permission of the IEEE Sensors Journal. (**c**) Deformation of the ring of the probe as an effect of the application of an external force.

In Figure 1, the architecture of the probe is presented, which is entirely composed of PDMS. The probe is designed to apply or detect a force in the axial direction (z-direction in the figure) through the spring-like deformation of the central ring, which is the most flexible and compliant section of the probe. We reported a thorough finite element (FEM) analysis of the behavior of the probe when loaded by a force [40,41]. As discussed in those works, a typical deformation of the ring is expected, due to the interaction with a target, such as a hair cell bundle (Figure 1b), as seen in Figure 1c. For the simulations, PDMS was modelled as a hyperelastic material [40] to take into account its nonlinear behavior under large deformation loads. Moreover, a Young’s modulus of 750 kPa was obtained for a 10:1 mixing ratio between the base and curing agent, which was chosen to characterize the elastomer as a soft material [42]. In particular, we used PDMS as the material of the probe because of its excellent physical properties [43], which include temperature stability, flexibility, chemical inertness, biocompatibility [44], elasticity and optical transparency in the visible spectrum [45,46]. In the FEM model, the force is applied at the tip in the z-direction, producing the deformation of the ring, while the pad is constrained. In the linear range of small deformation loads, the applied force is the tip displacement times the ring stiffness (Hooke’s law). However, for the experimental use, the probe was driven by a piezoelectric actuator to which it was clamped by means of a thick metallic wire glued onto the pad of the probe. The metallic wire was attached to the actuator by a custom-made holder. The measurement system, globally composed of the piezo-actuator, holder, wire and probe, was mounted under an upright microscope. Probe movements, namely the ring deformations, were optically observed by means of a camera or a photodiode on the microscope phototube. So, if the stiffness of the probe is known, the device in contact with a hair bundle can work as an actuator by applying voltage to the piezoelectric actuator and by moving the pad in the z-direction. As an effect of the interaction with the target the ring is deformed in the same direction and the displacement of the probe tip can be optically detected by means of the microscope. The applied force is the differential motion between the pad and the tip multiplied by the stiffness. Conversely, when the probe detects forces generated by the target, such as the active forces produced by a hair bundle, which provides nanometer scaled deformations, the probe works as a sensor. Measurements are only reliable if the probe tip and the target perfectly fit after the engagement. Therefore, the end geometry of probes should be properly shaped to fit the target as precisely as possible. For fitting hair bundles, a V-shaped tip was chosen, since the bundles are usually characterized by this typical profile (see Figure 1b). In particular, the bundle in the figure is a mammalian outer hair cell, composed of three rows of rod-like stereocilia of different heights (ranging from 2 to 5 μm), forming a V-shaped profile with wing length *W*_L_ ≈ 4 μm, total width *W*_E_ ≈ 6 μm and pitch angle α = 60°÷95°. These geometric characteristics can also vary along the cochlea, so the design of probes should take into account the shape of cells and their geometric variability. In particular, the tip arm width of the structures was fixed equal to *W*_E_ = 10 μm, while the lengths of the tip wings changed with the specific angle pitch.

Knowing the stiffness of the probe under use is a key point for reliable sensing. The stiffness depends on both the structural properties of the materials and geometrical parameters. In particular, from a geometrical point of view, the stiffness of the probe is proportional according the following equation:

*k* ∝ 1/(*R*_M_)^3^ (1)

where

*R*_M_ = ⅟₂ (*R*_in_ + *R*_out_) (2)

is the ring’s medium radius and *R*_in_ and *R*_out_ are the inner and outer radii of the ring, respectively (Figure 1a). *R*_in_ and *R*_out_ can be accurately tuned for the control of the compliance. However, for reproducible fabrication, the ring width (i.e., the difference between radii) is left unchanged and equals to *R*_out_ − *R*_in_ = 7 μm. The crucial role of the ring is evidently highlighted when observing Figure 1c, as it can be seen that it is the most stressed component of the probe, where larger deformations occur, especially in correspondence with its lateral extremities. As an effect of the deformation of the ring, the tip arm is the component of the probe is displaced more, whereas the parts of the probe closer to constraints, namely the base arm and pad, are hardly affected by sensitive displacements. Moreover, the thickness of the probe is also critical for the tuning of the stiffness, and even for the success of fabrication and the driving of the probe in the experiments. Considering that stereocilia are not thicker than 5 μm, to have a good fitting and permit the visual observation of the cell during excitation, probes should not exceed that limit. Consequently, the thickness of the fabricated probes was set equal to 8.5 μm.

**Figure 2:**
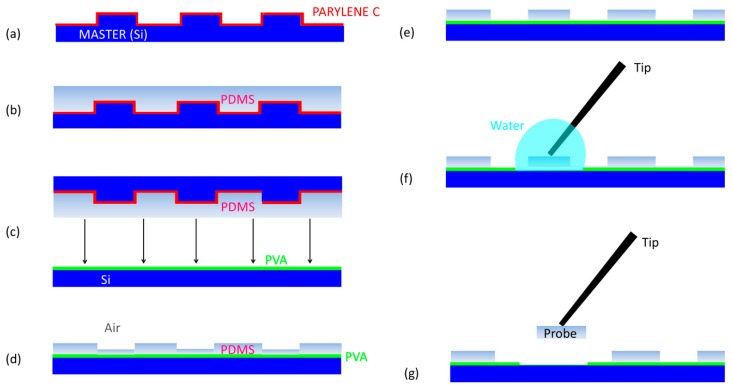
Workflow of the fabrication processing: (**a**) Master for molding and Parylene C coating; (**b**) PDMS spin coating on master; (**c**) thermal imprinting lithography; (**d**) demolding of the master; (**e**) removal of the PDMS residual layer; (**f**) metal wire tip gluing and (**g**) lift-off process.

PDMS probes are fabricated by thermal imprinting lithography (TIL) micromachining technology (Eitre 3, Obducat, Lund, Sweden), combining usual and unconventional lithographic methods. The fabrication processing is schematically illustrated in Figure 2. The first part of the processing, seen in Figure 2a, is the fabrication of masters for molding, which are made in silicon (Si) using standard optical lithography and dry etching techniques. A conformal layer of Parylene C is deposited on stamps at the end of lithography as anti-sticking promoter.

**Figure 3:**
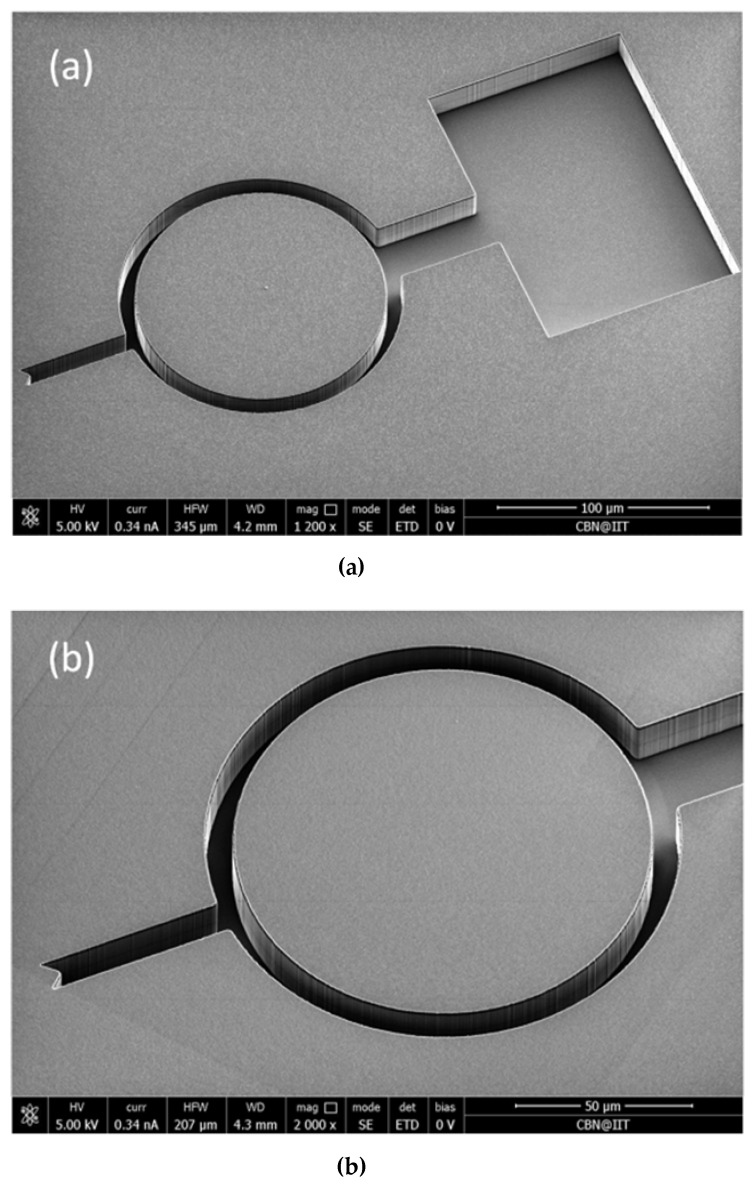
Scanning electron microscopy (SEM) images of a Si master obtained by means of the Bosch method. (**a**) Entire probe (*R*_M_ = 46.5 μm, *T* = 12 μm). (**b**) Detail of ring and V-tip.

Masters for imprinting were fabricated on 390 μm-thick double polished silicon substrates. The optical lithography provided the probe pattern, which was then transferred to the Si by deep reactive ion etching (DRIE). Since the probes are high-aspect ratio structures, a “Bosch” DRIE process was performed using induced coupled plasma (ICP, Oxford Estrelas, Oxford Instruments, Abingdon, UK) to accomplish a directional and quite deep vertical etching with lateral selectivity and optimized sidewalls roughness. In the Bosch procedure, a sequence of passivation and etching slots was alternatively switched when using the ICP. In the passivation time slot (500 ms), a polymer coating of C_4_F_8_ was deposited on the surfaces and sidewalls for protection [47], with a gas flow rate of 140 sccm, a chamber pressure of 20 mTorr and a coil power of 1400 W (*f* = 13.56 MHz). Then, in the etching time slot (700 ms), the Si was etched by a SiF_6_ gas flow of 200 sccm, generated by a coil power of 1500 W (*f* = 13.56 MHz) at a pressure of 30 mTorr, with a bias power of 25 W (*f* = 500 Hz) for increased anisotropy [48]. Finally, the processing was optimized to fabricate masters with scallops of only 100–120 nm. The depth of etching (*T*) was 12 μm. In Figure 3, scanning electron microscopy (SEM, Nova NanoSEM 200, FEI, Hillsboro, OR, USA) images of a master are shown. Figure 3a shows the entire view of the probe, whereas Figure 3b details the ring and the V-tip. Even though the final thickness of the probe (*T*) was 8.5 μm, the masters were thicker to take into account the further steps of processing, namely the Parylene C deposition and the critical removal of the PDMS residual layer, which were expected to reduce the thickness of the imprinted probes by up to 3.5 μm.

The Bosch-etched Si masters (type 100, supplied by Okmetic, Vantaa, Finland) were coated by approximately a 1 μm-thick conformal layer of Parylene C (purchased as dimer powder from Specialty Coating Systems, Indianapolis, IN, USA), which was deposited using chemical vapor deposition (CVD, PDS 2010 Lab- coater system model, Specialty Coating Systems). Parylene C was used as an anti-adhesion interface for the PDMS replica during the de-molding at the end of the thermal imprinting step. As this polymer is widely appealing in many micro-patterning applications due to its optical transparency, chemical inertness, biocompatibility and non-degradability [49,50], this polymer was chosen for its hydrophobicity (high contact angle and low surface energy) [51], which is able to weaken the strong adhesion of the thermally imprinted PDMS to Si. In addition, the deposition of Parylene C contributes to compensating the scallops on the sidewalls, providing as vertical of a profile as possible.

For the lithography of the PDMS probes, a 10:1 (w/w), 20:1 (w/w) and 30:1 (w/w) ratio of PDMS pre-polymer and curing agent (SYLGARD 184 Silicone Elastomer, Dow, Midland, MI, USA), respectively, was thoroughly mixed and degassed in a vacuum to remove bubbles. Then, it was spin-coated on the masters at 5000 rpm for 40 min (Figure 2b). A sacrificial layer of polyvinyl alcohol (PVA, Sigma-Aldrich, St. Louis, MO, USA) was deposited on the silicon substrate (Figure 2c). PVA will promote the lift-off of probes at the end of fabrication with water. PVA is a synthetic hydrophilic polymer, characterized by excellent chemical resistance, biodegradability, non-toxicity, solubility in water and insolubility in organic solvents [52]. For this application, a filtered solution of 24 wt.% PVA powder was dissolved in the water, deposited by spin coating on silicon wafers (2000 rpm, 40 min), then baked at 110 °C for 5 min to completely evaporate the water and leave a final sacrificial layer of ~3 μm. The thickness of the PVA is very critical in the fabrication processing, since too thin of a film (<2 μm) is not enough to detach the probes from the substrate, while a too thick film (>4 μm) cannot be fully dissolved by a drop of water.

TIL consists in two steps. In the first one, the master with the spin-coated PDMS is pressed on the Si substrate with the prescribed temperatures and pressure by a thermal imprinter (Figure 2c). During the processing, an initial heating time frame (90 °C for 15 min) was used, which is useful to promote the PDMS filling the template cavities and curing. This was followed by a second cooling time frame (40 °C for 5 min), in which the PDMS strain, induced by pressure, was relaxed. While performing these two temporal frames, the pressure was constant and kept equal to 10 bar. As an effect of this pressure, a force was equally distributed on the surface of the stack, such that the master and substrate tended to conform to each other and the effect of thickness variation was reduced. Then, the master and substrate were de-molded, but a PDMS residual layer remained (Figure 2d). In the second step, the residual layer of PDMS (~3 μm) is dry etched from the imprinted area of the substrate. Since PDMS is a silicon-based polymer, for the etching process, a SF_6_/O_2_ (10/2 sccm) chemistry at a working pressure of 7 mTorr and a coil power of 1000 W was exploited. In addition, the DC bias was 170 V, (platen RF power 150 W at 13.56 MHz) and *T* = 50 °C (Figure 2e).

**Figure 4:**
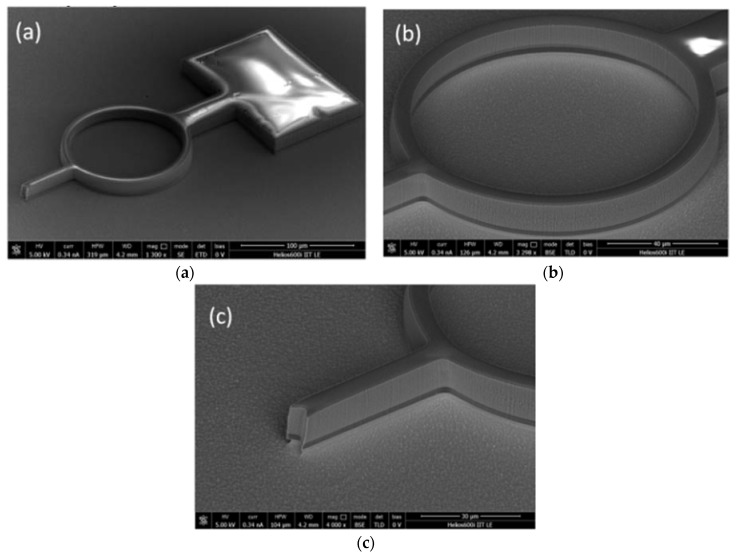
SEM images of the result of the thermal imprinting lithography of a PDMS probe. In the image, the darker layer underneath the PDMS profile of the probe is the PVA film. (**a**) Entire probe; (**b**) detail of the ring; (**c**) detail of the V-shaped tip.

In Figure 4, SEM images of the final result of the TIL are shown, emphasizing the whole probe in Figure 4a and the details of the ring and V-shaped tip in Figure 4b,c, respectively. The probe represented in the figure is characterized by an *R*_M_ of approximately 46.5 μm (*R*_out_ = 50 μm, *R*_in_ = 43 μm) and *T* is approximately 8.5 μm. What is noteworthy is that the film of PVA underneath the profile of PDMS probe is clearly visible in the images, as that darker layer is at the base of probe.

Finally, the probes were lifted from the substrate by dissolving the PVA film with water after gluing a thick metal wire or strut to the pad of the probe (Figure 2f). The PVA sacrificial layer of ~3 μm, in combination with the exploitation of the counteracting force applied by the glued thick metal wire during the lift-off process, is enough to prevent any stiction problem being experienced by the probe (Figure 2g).

Tests of mechanical characterization to measure stiffness were performed by means of a commercially available silicon-based sensing probe (FT-S100 Microforce, Femto Tools), which was interfaced to a PC by a standard USB connection, and dedicated software (FT-WS01, Femto Tools) for real-time visualization and post-processing. The sample was mounted under an optical microscope on a Melles Griot 17MAX301 NanoMax-TS piezo-actuated stage for the nanometric positioning. The piezo-actuated NanoMax was used together with the Melles Griot piezoelectric actuator control module 17MPZ001 for modulating the position of the NanoMax stage in the three axes.

## 3. Results

**Figure 5:**
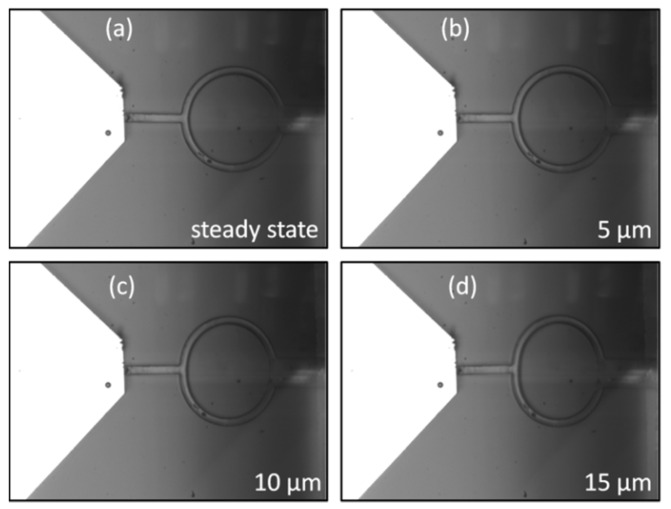
(**a**) Engagement between the Si tip of the FT-S100 sensor and a PDMS soft probe where *R*_M_ = 45.3 μm (*R*_out_ = 48.9 μm, *R*_in_ = 41.7 μm). Loading of the PDMS soft probe by driving a displacement of (**b**) 5 μm, (**c**) 10 μm and (**d**) 15 μm. These large displacements were chosen to help visualize the deformation of the ring during the interaction of Si probe with the PDMS probe, no matter the working regime.

The work of probes as force sensors or force actuators is based on the application of the linear Hooke’s law and on the experimental measurement of their stiffness. In the measurement setup, the sample is composed of a detached PDMS probe mounted on a Si support with a constrained pad and free tip and ring. This was done to mimic the experimental use, where only the tip interacts with the target and the pad is attached to the piezo-actuator through a metal wire. By driving the piezoelectric controller, the PDMS probe and the silicon probe sensor are primarily aligned and engaged; then the PDMS probe is moved against the stiffer tip of the Si sensor with the prescribed steps of displacement, which compressively load the V-shaped tip. In Figure 5, the engagement with the Si sensor (Figure 5a) and the loading of the PDMS probe with an imposed displacement of 5 μm (Figure 5b), 10 μm (Figure 5c) and 15 μm (Figure 5d), respectively, is shown. The probe in the image is characterized by *R*_M_ = 45.3 μm (*R*_out_ = 48.9 μm, *R*_in_ = 41.7 μm) and a pitch angle where α is ~80°. As evidently seen in the image, as a consequence of the application of a controlled motion, the softer ring of the probe deforms by shrinking axially and swelling transversally because of the applied force. For each displacement, the punctual stiffness is the force divided by the corresponding displacement in accordance with the definition of stiffness (Hooke’s law). It is worth emphasizing that PDMS is a nonlinear material, so the linear Hooke’s law is applicable only when the soft probe works in the small deformation regime (linear behavior) [41]. For a range of forces linearly dependent on displacements, the stiffness of a probe is calculated by measuring the angular coefficient of the force-displacement curve. For mechanical characterization, a set of probes with different geometric features, namely rings and thicknesses, was fabricated. Then, several proportions of base-curing agent in the preparation of the PDMS, namely 10:1, 20:1 and 30:1 (w/w), were explored for testing the effect on stiffness.

In Table 1, the ring sizes of probes made for testing and their experimental stiffnesses are reported. These data refer to PDMS probes with a 10:1 (w/w) base-curing agent proportion. Each size was measured by means of a scanning electron microscope (SEM). The thickness of the probes (*T*) was 8.5 μm.

**Figure 6:**
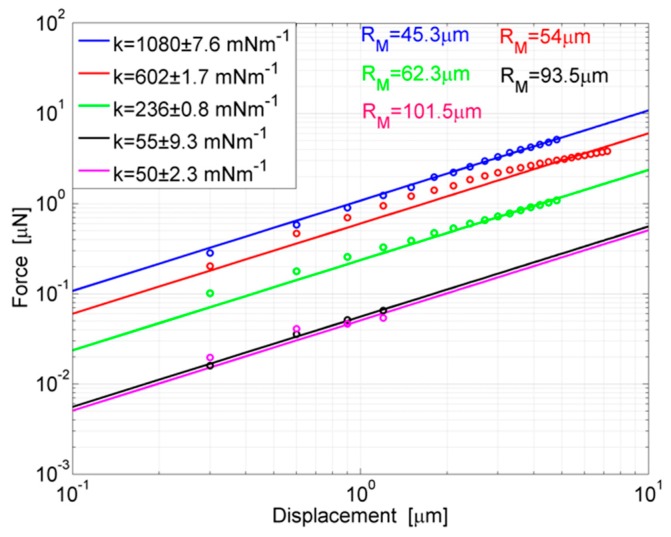
Force-displacement regression lines and linear data for probes in Table 1. Measurements are repeated three times and averaged data are reported in the plot. For each probe the stiffness is the angular coefficient of the regression line.

Force-displacement regression curves and the linear data are reported in Figure 6 for the probes of Table 1 to highlight the progressive reduction of stiffness experienced by devices with larger rings. For each probe, the force-displacement curve was calculated by increasing the applied displacement by steps of 300 nm and measuring the corresponding force by sensing. A repetition of three measurements was carried out for each probe and each point is the mean value of three experimental acquisitions. Curves in the figure show the reversible linear behavior which occurs for small displacements. For increasing steps of loading, the curves tend to diverge with a nonlinear trend.

For silicones such as PDMS, different from other materials, the nonlinear region may still be as reversible as the linear one, however, it already arises for small deformations of the ring, in dependence on its compliance. For example, the less compliant probe, where *R*_M_ = 45.3 μm, is out of the linear behavior for an applied force (*F*) of ~5.5 μN; the probe where *R*_M_ = 62.3 μm for a force of ~1.1 μN while one of the most compliant probes, *R*_M_ = 93.5 μm, for a force of ~80 nN. However, the linear section of the force-displacement curve is the useful region for stiffness measurements. So, a regression curve of measurement points in the linear region was calculated and the angular coefficient was defined as the stiffness. From the observation of curves, it is worth noting how the stiffness tends to decrease more and more slowly for progressively larger rings. For instance, an enlargement of ring from *R*_M_ = 45.3 μm to *R*_M_ = 54 μm determines a reduction of stiffness of ~478 mNm^−1^, whereas an identical variation from *R*_M_ = 93.5 μm to *R*_M_ = 101.5 μm only provides a stiffness difference of ~5 mNm^−1^. This can be explained by considering that the stiffness of the probe follows Equation (1), so it varies very quickly until an asymptotic behavior is reached. For radii in that region, a dramatic extension of geometry is needed to obtain sensitive changes in stiffness.

**Figure 7:**
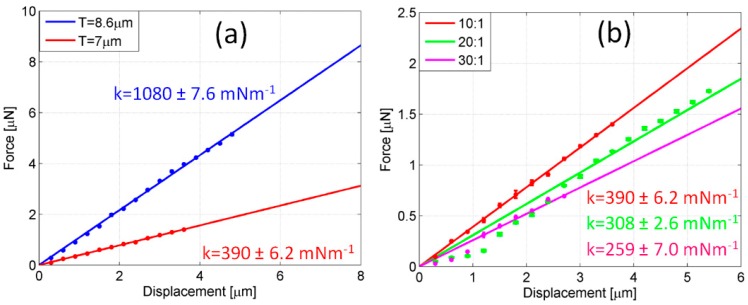
Effect of thickness and Young’s modulus on the stiffness of probes. (**a**) Comparison of stiffness between two equally ring-sized probes (*R*_M_ = 46.5 μm) and a fixed mixing ratio (10:1), but with different thicknesses: *T* = 8.5 μm (blue line) and *T* = 7 μm (red line). (**b**) Comparison of stiffness between three equally ring-sized (*R*_M_ = 46.5 μm) and thickness-sized (*T* = 7 μm) probes, but with different mixing ratios: 10:1 (red line), 20:1 (green line) and 30:1 (magenta line). Measurements were repeated three times and averaged data are reported in the plot. For each probe, the stiffness is the angular coefficient of the regression line.

Additionally, other parameters also have a direct effect on stiffness and could be involved for the improvement of compliance, such as the thickness and Young’s modulus. Figure 7 displays the effect of thickness and Young’s modulus on the stiffness of probes. In Figure 7a, the less compliant probe (*R*_M_ = 45.3 μm) which was previously tested is compared to an identical probe (max 10% of ring size variation), lifted off from the same chip, but made thinner through an extra plasma treatment. A final thickness of 7 μm was set. The mixing ratio between the oligomer and the curing agent was equal to 10:1. The measurement of stiffness revealed an impressive reduction of stiffness from k = 1080 ± 7.6 mNm^−1^ (blue line, *T* = 8.5 μm) to k = 390 ± 6.2 mNm^−1^ (red line, *T* = 7 μm), as a consequence of the linear dependence of stiffness on thickness. However, a further decrease of thickness is unsuitable as a strategy for lowering stiffness because of the increasing difficulty in detaching thinner probes. In Figure 7b, the results of thickness reduction and Young’s modulus tuning are combined.

In the plot of Figure 7b, the thinner probe used for comparison in Figure 7a, characterized by a 10:1 mixing ratio and *T* = 7 μm (red line), is replicated (max 10% of variation of ring and thickness size) by means of the same master, but with mixing ratios equal to 20:1 (green line) and 30:1 (magenta line), respectively. The amount of curing agent mixed with the base is expected to play a crucial role in the final hardness of the cured polymer. The cross-linker connects the PDMS chains and a higher concentration of curing agent leads to a larger number of chain links, resulting in a more rigid cured structure [53]. Hence, a gradual reduction of curing agent probes exhibits an increased compliance, as highlighted in the figure, where the initial stiffness of the 10:1 probe is 390 ± 6.2 mNm^−1^ and drops off to 308 ± 2.6 mNm^−1^ and 259 ± 7.0 mNm^−1^ for the 20:1 and 30:1 probes, respectively. As already discussed, the enlargement of the central ring progressively reduces the stiffness of probes. However, according to Equation (1), the lowering of stiffness tends to be slowed down for ring sizes in the asymptotic regime. As a consequence, larger and larger rings are required for a modest decrease in stiffness, unless a variation of the PDMS mixing ratio is introduced.

**Figure 8:**
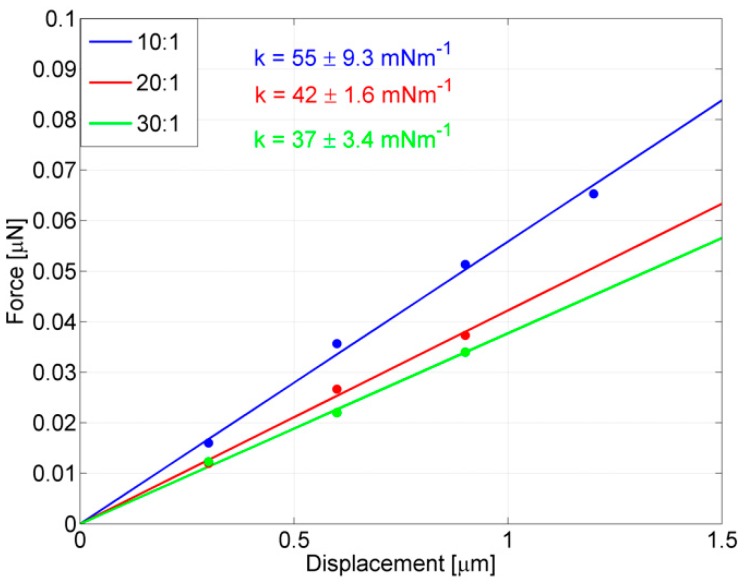
Stiffness measurements of one of the most compliant probes where *R*_M_ = 93.5 μm and *T* = 8.5 μm, fabricated with different Young’s moduli, tuned by dosing the PDMS mixing ratio (w/w). Ratios of 10:1 (blue line), 20:1 (red line) and 30:1 (green line), respectively, were used. Measurements were repeated three times and the averaged data are reported in the plot. For each probe, the stiffness is the angular coefficient of the regression line.

In the perspective of also using these probes for stimulating targets dealing with lower and lower forces, the crucial contribution of chemical tuning, due to changing the PDMS mixing ratio for further reducing the stiffness in probes with larger rings, is stressed in Figure 8, where the effect of tuning Young’s modulus on one of the most compliant probes (*R*_M_ = 93.5 μm) is shown. The probe was replicated (max 10% of ring size variation) by means of the same master, the thickness was set to 8.5 μm (max 10% of variation) and the PDMS mixing ratios were set equal to 10:1 (blue line), 20:1 (red line) and 30:1 (green line), respectively. The stiffness of the 10:1 probe, already tested above as 55 ± 9.3 mNm^−1^, and a further enlargement of the ring barely improved the compliance of the device (*k* = 50 ± 2.3 mNm^−1^ is the stiffness obtained for *R*_M_ = 101.5 μm by only varying geometrical parameters), while the 20:1 probe has a stiffness of 42 ± 1.6 mNm^−1^ and the 30:1 probe has a stiffness of 37 ± 3.4 mNm^−1^, which is the most compliant probe obtained in this work.

## 4. Discussion

The goal of this work was to design, fabricate and characterize an innovative force delivery and sensing probe for the study of biological processes involving force-dependent mechanisms, such as mechanotransduction. Depending on the species and organs of interest, the forces are in the piconewton to micronewton range, with time responses of microseconds and characteristic frequencies up to 100 kHz. On the basis of a preliminary FEM study, the fabrication processing and measurement characterization of a tunable ring-spring PDMS probe that is able to cover a very large range of compliances was developed. The probes were processed by means of a TIL micromachining system with silicon stamps, coated by Parylene C as an anti-adhesion promoter, and imprinted silicon substrates coated by PVA as sacrificial layer. An etching processing was then performed to remove the residual layer of PDMS from the imprinted area. Before final use, the probes were lifted from the substrate by dissolving the PVA with water. The experimental characterization of the real stiffness of PDMS probes was investigated by a silicon-based micro-force sensing probe. In particular, by tuning the geometrical size of the central ring from a medium radius (45.3 μm) to 101.5 μm, a stiffness ranging from 1080 ± 7.6 mNm^−1^ to 50 ± 2.3 mNm^−1^ was obtained for a PDMS mixing ratio equal to 10:1 (w/w) and thickness of 8.5 μm. A larger stiffness is easily attainable by reducing the ring size, whereas a lower stiffness can be set by making the probes thinner and/or changing the base-curing agent proportion during the preparation of the PDMS. If the thickness is varied, the stiffness of a probe with *R*_M_ = 45.3 μm, fabricated with a 10:1 mixing ratio (w/w) and a thickness where *T* = 7 μm, dramatically drops off from 1080 ± 7.6 mNm^−1^, obtained for *T* = 8.5 μm, to 390 ± 6.2 mNm^−1^. Finally, if chemical tuning is introduced, a probe with *R*_M_ = 93.5 μm, characterized by a stiffness of 55 ± 9.3 mNm^−1^ for a mixing ratio 10:1 (w/w) and *T* = 8.5 μm, is fabricated by means of a less-cured PDMS (ratios of 20:1 (w/w) and 30:1 (w/w)), the measured stiffness drops to 42 ± 1.6 mNm^−1^ and 37 ± 3.4 mNm^−1^, respectively. These compliances, combined with a proper design of the tip geometry, can open up new possibilities for the delivering and sensing of forces in the study of mechanosensitive mechanisms, which play a role for embryogenesis, vascular physiology and in a variety of sensory systems, both in vertebrates and invertebrates.

## Figures and Tables

**Table 1:** Geometrical ring sizes of the PDMS (10:1, w/w) soft probes fabricated for mechanical tests and their measured stiffnesses. The thickness of the probes (*T*) was 8.5 μm.

*R*_M_ [μm]	*R*_out_ [μm]	*R*_in_ [μm]	k [mNm^−1^]
45.3	48.9	41.7	1080 ± 7.6
54	57	51	602 ± 1.7
62.3	65.5	59	236 ± 0.8
93.5	97	90	55 ± 9.3
101.5	105	98	50 ± 2.3
